# Lymphoid tissue residency: A key to understand Tcf-1^+^PD-1^+^ T cells

**DOI:** 10.3389/fimmu.2022.1074698

**Published:** 2022-12-07

**Authors:** Chaoyu Ma, Nu Zhang

**Affiliations:** Department of Microbiology, Immunology and Molecular Genetics, Long School of Medicine, University of Texas Health Science Center at San Antonio, San Antonio, TX, United States

**Keywords:** TGF-beta, TCF-1, tissue-resident, chronic infection, tumor, lymph node

## Abstract

During chronic antigen exposure, a subset of exhausted CD8^+^ T cells differentiate into stem cell-like or progenitor-like T cells expressing both transcription factor Tcf-1 (T cell factor-1) and co-inhibitory receptor PD-1. These Tcf-1^+^ stem-like or progenitor exhausted T cells represent the key target for immunotherapies. Deeper understanding of the biology of Tcf-1^+^PD-1^+^ CD8^+^ T cells will lead to rational design of future immunotherapies. Here, we summarize recent findings about the migratory and resident behavior of Tcf-1^+^ T cells. Specifically, we will focus on TGF-β-dependent lymphoid tissue residency program of Tcf-1^+^ T cells, which may represent a key to understanding the differentiation and maintenance of Tcf-1^+^ stem-like CD8^+^ T cells during persistent antigen stimulation.

## Introduction

During acute antigenic exposure, such as acute viral infections or vaccination, naïve CD8^+^ T cells are activated by professional antigen presenting cells carrying cognate antigenic peptide/MHC-I complex in secondary lymphoid organs. Activated T cells undergo massive proliferation and further differentiate into effector CD8^+^ T cells with profound alterations in effector molecule production, migration pattern, transcriptional network, metabolic program, and epigenetic landscape. Shortly after antigen clearance, effector T cells undergo contraction and further differentiation into long-lived memory T cells with superior recall capacity (1). However, when antigen presence is prolonged (such as chronic infection and tumor), effector T cells rapidly turn to a different path towards exhaustion with greatly reduced effector function and population size. In recent decades, reviving exhausted T cells have been established as one of the common goals in tumor immunotherapies (2). Thus, it is essential to advance our understanding of exhausted T cells. Here, we will summarize recent findings related to the migration and tissue residency of a subset of exhausted T cells expressing transcription factor Tcf-1 (T cell factor-1).

## Tcf-1^+^ stem-like T cells

Shortly after the discovery of T cell exhaustion, it has been realized that exhausted T cells are not homogenous. Instead, a broad spectrum of T cell subsets together constitute exhausted T cell population. Initially, different exhausted T cell subsets were distinguished by various levels or composition of inhibitory receptors (3, 4). Later, different exhausted T cell subsets with various expression of T-box transcription factors T-bet and Eomes levels were discovered (5). More recently, Tcf-1^+^ subset of exhausted T cells has been defined as the progenitor or stem-like subset to sustain the whole exhausted T cell population (6–10) (**Box 1**). Tcf-1^+^ cells further differentiate into transitional subsets (e.g., CX3CR1^+^ cells) as well as terminally exhausted cells (e.g., CD101^+^ cells) (11–13). Most importantly, Tcf-1^+^ subset is the one responding to PD-1 or PD-L1 blockade in both chronic viral infection and tumor settings (7, 9, 14). Further, Tcf-1^+^ exhausted T cells are the main target of therapeutic tumor vaccines (15). Together, Tcf-1^+^PD-1^+^ stem-like or progenitor exhausted T cells are the key CD8^+^ subset which can be self-sustained and further differentiate into other exhausted T cell populations.

Interestingly, a recent paper has further defined a subset of Tcf-1^+^PD-1^+^ T cells carrying T_CM_ (central memory T cells) marker CD62L and CCR7, which is highly enriched for stem-cell or progenitor activity and largely responsible for PD-1 blockade induced T cell expansion (16). These Tcf-1^+^PD-1^+^CD62L^+^ stem-like CD8^+^ T cells is critically dependent on transcription factor Myb, which reminds us about a similar Myb-dependent CD8^+^ T_CM_ subset generated after acute viral infection (17). Accumulating evidence has documented the similarity between T_CM_ cells generated after acute infection (18) and stem-like exhausted T cells during chronic antigen exposure, especially regarding the Tcf-1-dependent genetic signature. At molecular level, it has been recently demonstrated that during memory T cell recall responses, there are a large collection of immediate responsive genes, including glycolytic enzymes, cell cycle controllers and transcriptional regulators. Tcf-1 is essential to keep these genes ready for future recall response *via* maintaining their 3D genomic interaction with distal enhancers (19). Consistent with this role of Tcf-1 in memory T cells, Tcf-1 and closely related transcription factor Lef-1 controls the 3D structure and crosstalk between distal genomic elements partially *via* interacting with CTCF (CCCTC-binding factor) in naïve T cells (20, 21). Thus, it is safe to conclude that Tcf-1-control transcription and epigenetic programs represents one of the central themes for most T cells (e.g., naïve, T_CM_ and stem-like) with robust expansion and differentiation capacity.

Box 1 **CD8+ T cell subsets.**

After acute infection, memory CD8^+^ T cells can be classified into three main subsets based on their migration pattern. T_CM_s (central memory T cells) carry lymph node homing receptors CCR7 and CD62L, and circulate via spleen, lymph nodes, blood and lymph. T_EM_s (effector memory T cells) lack lymph node homing receptors and circulate via spleen, blood and peripheral non-lymphoid tissues. T_RM_s (tissue-resident memory T cells) may carry tissue-resident markers, e.g., CD69^+^ and CD103^+/-^, and are non-circulating.

During chronic antigen exposure, exhausted CD8^+^ T cells can be classified into Tcf-1^+^ progenitor or stem-like T cells and Tcf-1^-^ T cells. The migration and residency of Tcf-1^+^ T cells are the focus of the current review. Based on migration pattern, Tcf-1^-^ CD8^+^ exhausted T cells can be further categorized into a migratory subset (i.e., CD69^-^CX3CR1^+^ and with superior effector function) and a resident subset (e.g., CD69^+^CD101^+^ and with diminished effector function).

## Tissue-resident memory T cells

Based on migration pattern, acute antigen exposure induced memory T cells can be categorized into central memory (T_CM_), effector memory (T_EM_) and tissue resident memory (T_RM_) T cells (22) (Box1). Because of the broad TCR repertoire, the frequency of T cells bearing TCR with a given specificity is often extremely low. To efficiently protect the whole body against potential antigenic evasion, continuous migration and patrolling for cognate antigen appearance is a build-in feature of T cell biology. Thus, the very existence of T_RM_ cells, which are largely separated from the circulation at steady states and confined to a specific tissue represents an intriguing “outlier”. Numerous efforts have been devoted to investigating the differentiation, molecular regulation and function of T_RM_ cells (23). T_RM_ cells are direct decedent of effector T cells. They often strategically located at previous pathogen entering sites or peripheral tissues experienced local inflammation and damage. In adult human and immunized animals, T_RM_ cells can be detected in most non-lymphoid tissues, including both mucosal and non-mucosal sites as well as the tissues which have been traditionally considered as immune-privileged sites (24–26). Number wise, T_RM_ represents the most abundant T cell population in most antigen-experience individuals.

Several local signals are actively involved in T_RM_ differentiation. For example, TNF (tumor necrosis factor), IL-33, extracellular ATP and local ICOS signals can promotes T_RM_ formation (27–30). Here, we will limit our discussion to two of the most well-studied signals for T_RM_ differentiation. First, we will focus on TGF-β (transforming growth factor-β), which is cytokine essential for CD103 (encoded by *Itgae*) induction on activated CD8^+^ T cells. CD103 is a commonly used marker for mucosal T_RM_s and critically involved in mucosal T_RM_ retention *via* interaction with its ligand E-cadherin (31–33). It is well established that TGF-β signal delivered to CD8^+^ T cells is broadly required for T_RM_ differentiation, including most mucosal T_RM_ with CD103 expression (31–34) and some non-mucosal T_RM_ lacking CD103 (35). Further, continuous TGF-β signal is required for long-term maintenance of T_RM_ cells in both skin and intestine (36, 37). Interestingly, dendritic cells deliver basal TGF-β to naïve T cells inside secondary lymphoid organs. This basal TGF-β signaling before T cell activation will keep naïve T cells semi-ready for later priming towards T_RM_ differentiation (38). Thus, during T_RM_ differentiation and maintenance, TGF-β signal is required at different locations and different stages. Similar to most dogmas in biology, the requirement for TGF-β in T_RM_ is not universal. Prominent exceptions do exist, i.e., T_RM_s isolated from upper respiratory tract and liver are formed independent of TGF-β signal following acute infection (39, 40). It is interesting to note that some TGF-β-dependent T_RM_ population carry higher levels of inhibitor receptor PD-1 expression (39, 41).

The second signal we would like to discuss here is local antigen. As T_RM_ is often formed at the site of local infection, which is likely associated with enhanced local antigen presentation. T_RM_ induction in the brain, the sensory ganglia, the lung and the cornea requires local antigen recognition (24, 25, 42, 43). However, local antigen is not essential for T_RM_ formation in the skin, the gut and the female reproductive tract (43–45). For example, chemical-induced local sterile inflammation can effectively attract *in vitro* activated CD8^+^ T cells to form skin T_RM_, which is a commonly used and convenient technique in T_RM_ field (43). However, even for skin T_RM_s, local antigen significantly boosts their formation (46, 47). After T_RM_ formation, it is generally believed that long-term maintenance of T_RM_ is TCR-independent, which is first demonstrated in skin-resident γδT cells (48), later confirmed in both CD8^+^ and CD4^+^ T_RM_s (49–51). Together, although not universally required, TGF-β and local antigen often promote initial T_RM_ formation. For long-term T_RM_ maintenance, TGF-β is likely involved while antigen is not required.

## T_RM_ in secondary lymphoid organs

Although initial CD8^+^ T_RM_ research was largely focused on non-lymphoid tissues, it was quickly realized that T_RM_ could form inside secondary lymphoid organs [i.e., spleen and lymph nodes (LN)] after systemic viral infection although the population size was small (52). In systemic LCMV (lymphocytic choriomeningitis virus) infection model, lymphoid organ CD8^+^ T_RM_ does not express CD103. They carry typical T_RM_ markers CD69^+^Ly6C^-^CD62L^-^ and core T_RM_ gene signature. Importantly, these secondary lymphoid organ T_RM_s are not migratory as demonstrated in parabiosis experiments (53). They are direct derivative of upstream non-lymphoid tissue T_RM_. In other words, non-lymphoid tissue T_RM_ re-activation leads to robust T_RM_ accumulation inside draining LNs (53). Consistently, pet store mice or “dirty” mice with a complicated exposure history to a broad collection of environmental pathogens carried significantly increased T_RM_ population in secondary lymphoid organs (53). In local influenza virus infection model, a significant population of CD69^+^CD103^+^CD8^+^ T_RM_ subset can be identified in lung draining LNs (54–56). Repetitive infection promotes LN T_RM_s (54) and CD8^+^ T cells carrying different TCR specificity exhibit distinct LN T_RM_ potential (56), suggesting a possible role of antigen in LN T_RM_ formation. However, antigen is not required for LN T_RM_ maintenance (55). Similar to systemic LCMV infection, LN T_RM_ is generated *via* retrograde migration from upstream lung T_RM_s during influenza viral infection (55). Functionally, these draining LN T_RM_ may represent an expanded local defense to reinforce the first line of T_RM_-dependent immunity at the upstream non-lymphoid tissues.

Interestingly, a large number of memory CD8^+^ T cells in human LNs and spleen carry typical T_RM_ markers CD69 and CD103 (57). In addition, a CD69^+^CD103^+^ CD8^+^ T cell subset has been identified in human tonsil and specific for Epstein Barr Virus (EBV) (58). The identity, migration and function of these human T cells remains a mystery. Based on the observation in mice (especially the results from dirty mice), it is conceivable that these CD69^+^CD103^+^ CD8^+^ T cells in human secondary lymphoid organs may contain a significant T_RM_ subset. Thus, CD8^+^ T_RM_ can form inside secondary lymphoid organs in both mouse and human. In mouse acute infection models, these LN T_RM_s are derived from upstream non-lymphoid tissue T_RM_s. In other words, they may have a travel history to periphery tissues before settling down in the draining LNs.

## Lymphoid residency of stem-like T cells—Chronic infection

In the original papers that discovered Tcf-1^+^PD-1^+^ subset during chronic LCMV infection, a few interesting features of Tcf-1^+^ stem-like T cells emerged. First, they are largely located inside secondary lymphoid organs (LNs or splenic lymphoid follicles). Second, they are almost absent in the peripheral blood (7). Demonstrated *via* parabiosis experiments, most Tcf-1^+^PD-1^+^ T cells are tissue-resident and largely separated from the circulation after the establishment of chronic LCMV infection (59). Incorporating T_RM_ marker CD69, both Tcf-1^+^ stem-like and Tcf-1^-^ effector subsets can be further divided into CD69^+^ and CD69^-^ populations. Importantly, both Tcf-1^+^CD69^+^ and Tcf-1^-^CD69^+^ subsets are excluded from the circulation and negatively enriched for circulating T cell gene signature (60). Tcf-1^+^CD69^+^ cells are largely located inside lymphoid follicles while Tcf-1^-^CD69^+^ ones are splenic red pulp-resident (60). These results demonstrate that during systemic chronic viral infection, a significant portion of exhausted CD8^+^ T cells acquire certain features of T_RM_ inside lymphoid organs. Based on these findings, it will be interesting to address the questions why Tcf-1^+^ stem-like CD8^+^ T cells prefers a lymphoid environment and whether the lymphoid-residency is functionally important for stem-like T cell differentiation or maintenance.

**Figure 1:**
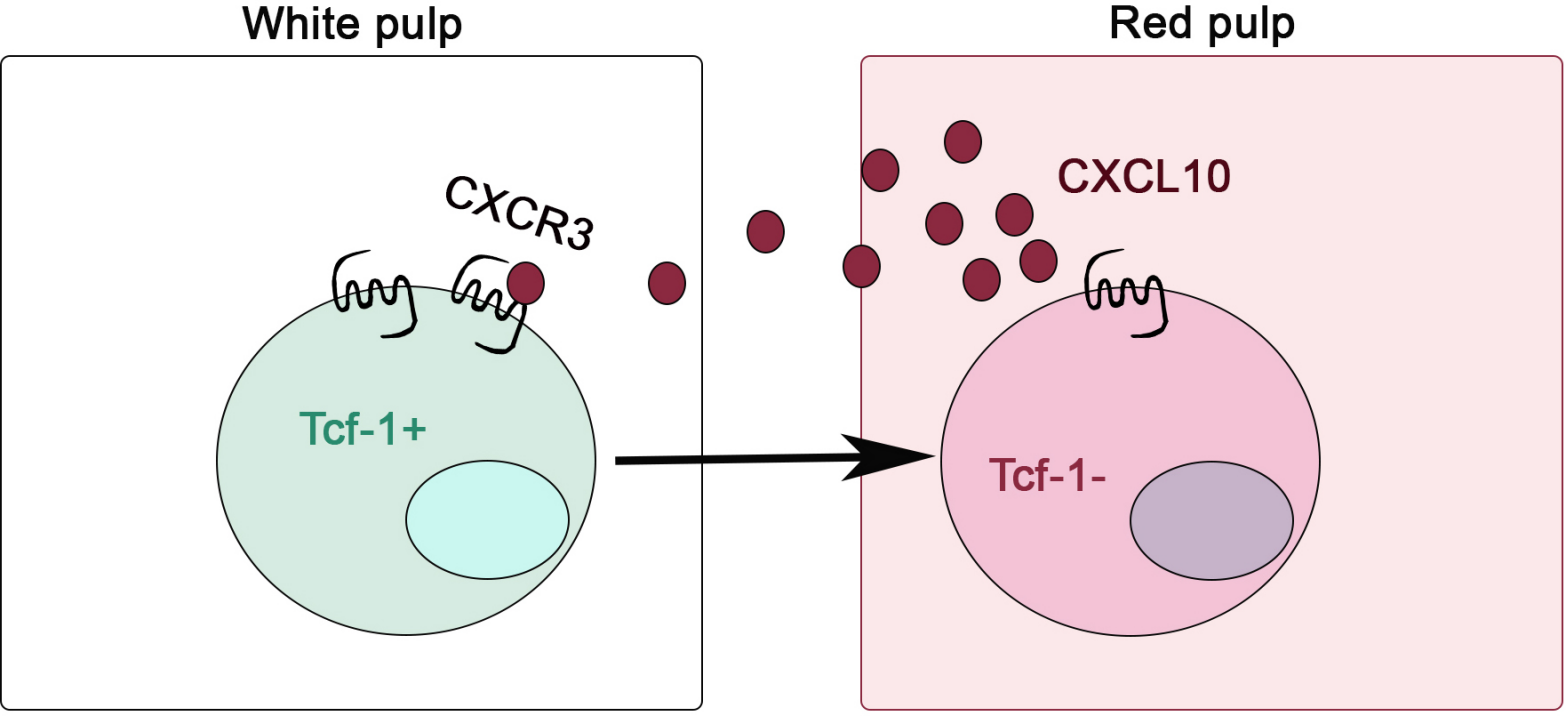
CXCR3 is critical for stem-like CD8^+^ T cells to leave lymphoid niche during chronic infection. CXCR3/CXCL10-dependent migration from splenic white pulp to red pulp is required for the efficient differentiation from Tcf-1^+^ stem-like to Tcf-1^-^ effector T cells.

A series of recent findings focused on chemokine receptor CXCR3 have shed light on these critical questions. Using either acute (61) or chronic LCMV infection model (62), it has been demonstrated that CXCR3 is essential for the differentiation from Tcf-1^+^ stem-like to Tcf-1^-^ effector T cells. In the absence of CXCR3, there is an increased accumulation of Tcf-1^+^ subset inside secondary lymphoid organs. There are two ligands for CXCR3 in C57BL/6 mice, namely CXCL9 and CXCL10. Interestingly, CXCL9 producing cells (e.g., XCR1^+^ cDC1) are concentrated inside T cell zone while CXCL10 producing cells (e.g., conventional Dendritic Cell 2, or cDC2 and inflammatory monocytes) are mainly outside T cell zone. Thus, it is mainly *via* CXCL10/CXCR3 interaction to attract Tcf-1^+^ T cells to move out of T cell zone (61, 62). These findings have been validated in a different chronic parasite infection model (i.e., *Toxoplasma gondii, or T. gondii* infection) (63). During *T. gondii* infection, Tcf-1^+^ CD8^+^ T cells expressing high levels of CXCR3. In responding to CXCL10, these stem-like T cells migrate out of lymphoid follicles and form clusters with cDC2 in the bridging channels of spleen. Importantly, these *T. gondii*-specific Tcf-1^+^CD8^+^ T cells isolated from the spleen carry a typical T_RM_ phenotype (i.e., *Cd69^+^Itgae^+^Klf2^-^S1pr1^-^S1pr5* ^-^) although this result is from RNA-seq, not confirmed at protein levels (63). Together, these investigations on CXCR3 and CXCL10 provide us an excellent example that the lymphoid location of Tcf-1^+^ stem-like T cells is tightly associated with their maintenance (**Figure 1**). Leaving lymphoid environment is accompanied by immediate effector differentiation.

Another key signal delivered to stem-like T cells is TGF-β. Although TGF-β is often considered as a cytokine with broad distribution, Tcf-1^+^ stem-like T cells carry TGF-β activating integrin (αvβ8) to keep a TGF-β-rich microenvironment around themselves (64). TGF-β is produced as inactive latent form. Active TGF-β has an extremely low solubility at neutral pH and therefore active TGF-β is likely to have a very short functional distance. Thus, local TGF-β-activating mechanisms (e.g., αvβ8 integrin) are essential for TGF-β function *in vivo*.

**Figure 2:**
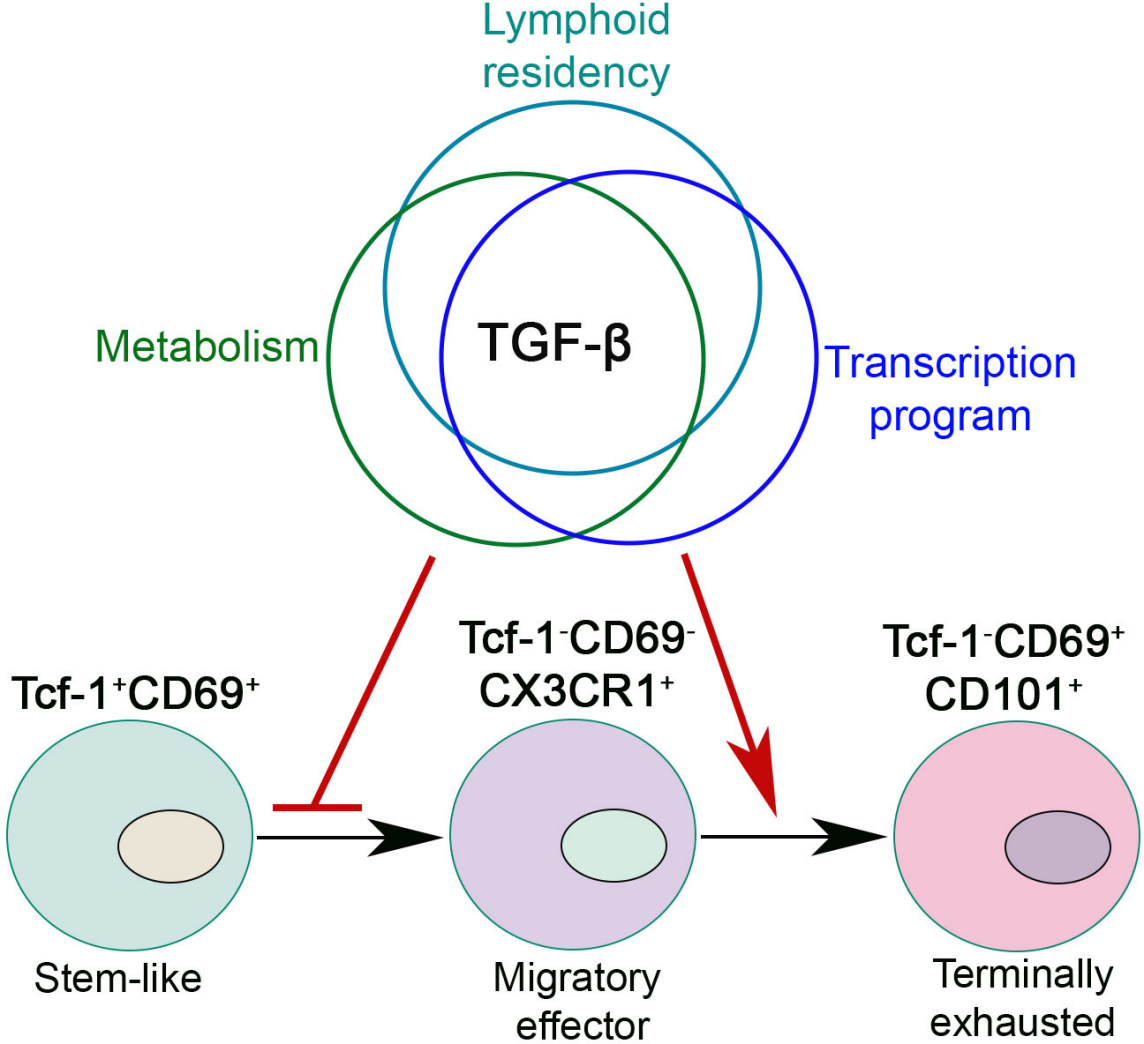
TGF-β controls exhausted CD8^+^ T cell differentiation during chronic viral infection. TGF-β integrates lymphoid residency, metabolic program and transcriptional control to inhibit the differentiation of migratory effectors and promote CD8^+^ T cell terminal exhaustion. In this figure, we present a lineal differentiation model for exhausted CD8^+^ T cells. To be noted, elegant evidence does exist to support a bifurcation model of exhausted T cell differentiation (65).

The function of TGF-β on stem-like T cells is multifaceted (**Figure 2**). First, TGF-β restrains mTOR (Mammalian Target of Rapamycin) activity in stem-like T cells to maintain their long-term responsiveness (64). Second, TGF-β directly suppress the differentiation of CX3CR1^+^ effector T cells and promotes the formation of CD101^+^ terminally exhausted T cells (64, 66, 67). Importantly, the impacts of TGF-β are significantly enhanced during the later stages of chronic infection (66). Finally, we have demonstrated that TGF-β suppresses Tcf-1^+^➔CX3CR1^+^ differentiation partially *via* enforcing their lymphoid tissue residency. In the absence of TGF-β receptor, stem-like T cells exhibited defective lymphoid tissue retention, which is associated with further effector differentiation. Forcing TGF-βR deficient stem-like T cells to stay inside lymphoid follicles *via* integrin α4 blocking partially corrects the defects. This result suggests that manipulating the location of Tcf-1^+^ T cells alone is sufficient to control their differentiation (67).

## Lymphoid residency of stem-like T cells—Tumor immunity

In tumor settings, Tcf-1^+^PD-1^+^ cells are initially identified among tumor infiltrating lymphocytes (TIL), which is out of a secondary lymphoid organ. It is later discovered that a lymphoid-like microenvironment exists inside solid tumors to host Tcf-1^+^ stem-like T cell subset and physically separates them from tumor cells (68, 69).

**Figure 3:**
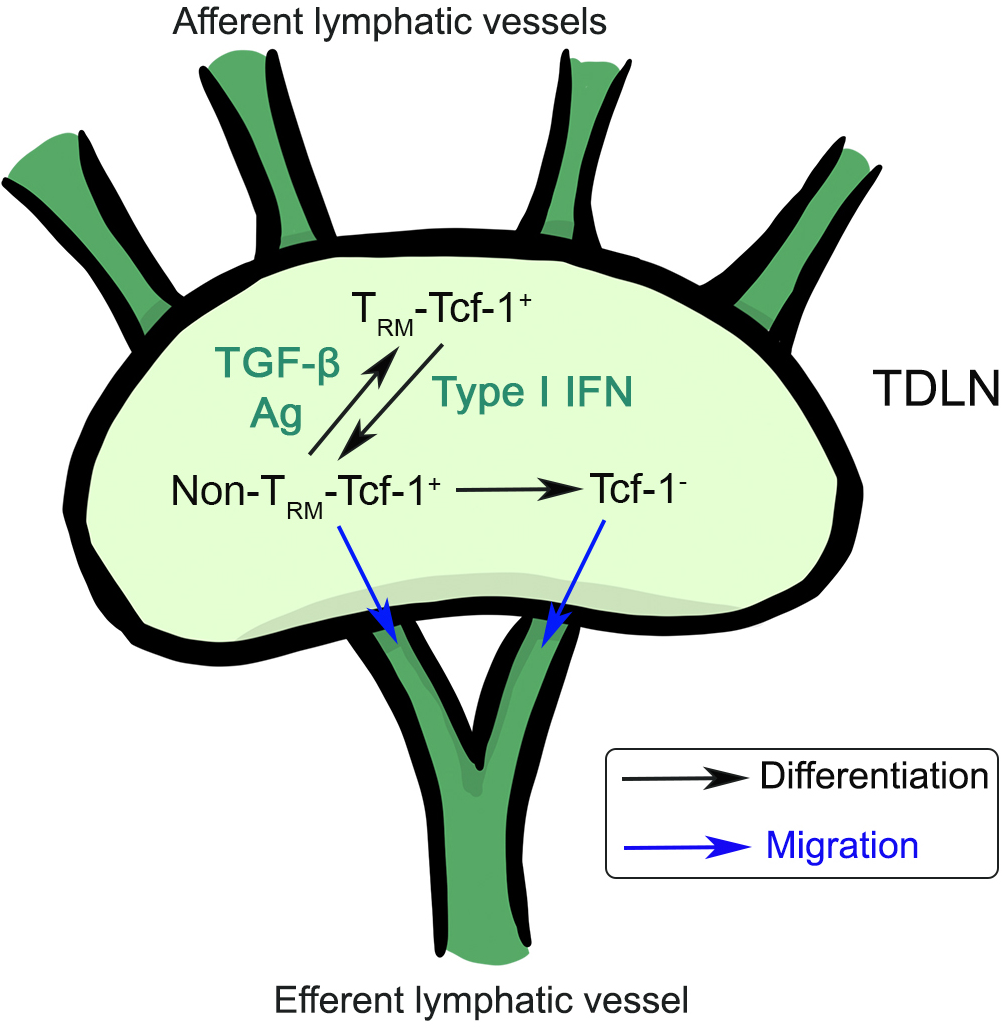
The differentiation and migration of stem-like CD8^+^ T cells inside tumor draining lymph nodes. T_RM_-Tcf-1^+^ cells can differentiate into non-T_RM_-Tcf-1^+^ cells, which can further differentiate into Tcf-1^-^ effector T cells. Non-T_RM_-Tcf-1^+^ and Tcf-1^-^ T cells have the capacity to migrate to distal organs. TGF-β, antigen and type I IFN control the differentiation of Tcf-1^+^ T cells inside tumor draining LNs.

Recent results have established cDC1-delivered tumor antigen is critical to establish tumor draining LNs as a reservoir of Tcf-1^+^ T cells and to sustain anti-tumor immunity (70, 71). Our recent work has revealed that tumor draining LN (TDLN) harbors a large population of Tcf-1^+^ CD8^+^ T cells with a CD69^+^CD103^+^ T_RM_ phenotype (72). The differentiation of T_RM_-Tcf-1^+^ T cells requires both TGF-β signaling and tumor antigen. Tumor vaccine, especially vaccine adjuvant promotes the differentiation from T_RM_ to non-T_RM_ in a type I IFN-controlled way. This result is consistent with the finding in an acute viral infection model, where type I IFN suppresses T_RM_ formation (35). The loss of T_RM_ feature is critical for the active migration of stem-like T cells from TDLN to tumor site to control tumor growth. In addition, the loss of T_RM_ identity may represent the first step of CX3CR1^+^ effector T cell differentiation. Another key finding is that Tcf-1^+^ CD8^+^ T cells gradually differentiate into T_RM_ inside TDLNs, i.e., the appearance of T_RM_-Tcf-1^+^ cells is significantly delayed comparing with that of Tcf-1^+^ cells in TDLNs. Only large tumor TDLN carries a significant population of T_RM_-stem CD8^+^ T cells. This finding likely explains the discrepancy between our results and most previous animal research focusing on early-stage tumor (i.e., when tumor is palpable). For example, in contrast to the lack of efficacy in our hands for large tumors, tumor vaccine is often effective when given early (15). Using photoconvertible mice, Tcf-1^+^ T cell migration between tumor and TDLN can be easily identified in early-stage tumor (when tumor size is small) (73). It is possible that similar to retrograde migration in acute infection settings, TDLN T_RM_-stem CD8^+^ T cells are derived from tumor infiltrating T cells although this idea has not been tested experimentally. Considering all these results, we believe that tumor-specific Tcf-1^+^CD8^+^ T cells accumulate inside TDLNs and gradually differentiate into T_RM_-stem and lose migratory capacity when tumor reaches a certain size (**Figure 3**). TGF-β and tumor antigen promote, while type I IFN inhibits the establishment of T_RM_-Tcf-1^+^ cells in TDLNs. It is conceivable that most cancer patients carry large tumors and likely harbor a significant portion on T_RM_-stem in TDLNs. The migration from TDLNs to tumor is essential for CD8^+^ T cells to directly attack solid tumors. Thus, targeting T_RM_-stem in TDLN and mobilizing TDLN stem-like CD8^+^ T cells will be one of the keys to boost tumor immunotherapies, including tumor vaccines.

## Conclusion and future

Together, lymphoid residency is an essential component of Tcf-1^+^ exhausted T cells in both chronic viral infection and tumor immunity. The regulation of lymphoid residency for Tcf-1^+^ T cells is critical to control effector differentiation and is an essential speed-limiting step for tumor vaccine response. However, T_RM_ is not the only fate for lymphoid Tcf-1^+^ exhausted CD8^+^ T cells. A significant portion of lymphoid Tcf-1^+^ CD8^+^ T cells does not differentiate into T_RM_. The regulation of T_RM_ vs non-T_RM_ Tcf-1^+^ T cells under different tumor immunotherapy settings remains unknown. The lineage relationship between T_RM_-Tcf-1^+^ vs non-T_RM_-Tcf-1^+^ cells is unclear. Importantly, whether T_RM_-Tcf-1^+^ T cells are critically involved in all chronic antigen exposure settings awaits future investigation. For example, in an autoimmune diabetes setting, pancreas draining LN Tcf-1^+^ CD8^+^ T cells do not carry enhanced CD69 and express high levels of *Klf2* (74), which is associated with circulating T cells (75). Similarly, in a melanoma and autoimmune vitiligo setting, LN Tcf-1^+^ T cells express high levels of *Klf2* and Tcf-1^-^ LN effector T cells become T_RM_ (76). Thus, it is possible that a unique mechanism exists to keep autoimmune-induced Tcf-1^+^ CD8^+^ T cells as circulating cells in lymphoid organs. Nevertheless, recent publications have highlighted the importance of the lymphoid location of Tcf-1^+^ T cells. Better understanding the control of residency vs migration of Tcf-1^+^ T cells represents one of the keys to advance our knowledge of Tcf-1^+^PD-1^+^ T cell biology and facilitate the future design of T cell-based immunotherapies.

## Author contributions

CM and NZ researched, wrote and edited the manuscript. All authors contributed to the article and approved the submitted version.

## References

[1] Williams MA Bevan MJ . Effector and memory CTL differentiation. Annu Rev Immunol (2007) 25:171–92. doi: 10.1146/annurev.immunol.25.022106.141548 17129182

[2] Hashimoto M Kamphorst AO Im SJ Kissick HT Pillai RN Ramalingam SS . CD8 T cell exhaustion in chronic infection and cancer: Opportunities for interventions. Annu Rev Med (2018) 69:301–18. doi: 10.1146/annurev-med-012017-043208 29414259

[3] Blackburn SD Shin H Freeman GJ Wherry EJ . Selective expansion of a subset of exhausted CD8 T cells by alphaPD-L1 blockade. Proc Natl Acad Sci U.S.A. (2008) 105:15016–21. doi: 10.1073/pnas.0801497105 PMC2567485 18809920

[4] Blackburn SD Shin H Haining WN Zou T Workman CJ Polley A . Coregulation of CD8+ T cell exhaustion by multiple inhibitory receptors during chronic viral infection. Nat Immunol (2009) 10:29–37. doi: 10.1038/ni.1679 19043418 PMC2605166

[5] Paley MA Kroy DC Odorizzi PM Johnnidis JB Dolfi DV Barnett BE . Progenitor and terminal subsets of CD8+ T cells cooperate to contain chronic viral infection. Science (2012) 338:1220–5. doi: 10.1126/science.1229620 PMC3653769 23197535

[6] He R Hou S Liu C Zhang A Bai Q Han M . Follicular CXCR5- expressing CD8(+) T cells curtail chronic viral infection. Nature (2016) 537:412–28. doi: 10.1038/nature19317 27501245

[7] Im SJ Hashimoto M Gerner MY Lee J Kissick HT Burger MC . Defining CD8+ T cells that provide the proliferative burst after PD-1 therapy. Nature (2016) 537:417–21. doi: 10.1038/nature19330 PMC5297183 27501248

[8] Leong YA Chen Y Ong HS Wu D Man K Deleage C . CXCR5(+) follicular cytotoxic T cells control viral infection in b cell follicles. Nat Immunol (2016) 17:1187–96. doi: 10.1038/ni.3543 27487330

[9] Utzschneider DT Charmoy M Chennupati V Pousse L Ferreira DP Calderon-Copete S . T Cell factor 1-expressing memory-like CD8(+) T cells sustain the immune response to chronic viral infections. Immunity (2016) 45:415–27. doi: 10.1016/j.immuni.2016.07.021 27533016

[10] Wu T Ji Y Moseman EA Xu HC Manglani M Kirby M . The TCF1-Bcl6 axis counteracts type I interferon to repress exhaustion and maintain T cell stemness. Sci Immunol 1 (2016) 1. doi: 10.1126/sciimmunol.aai8593 PMC5179228 28018990

[11] Zander R Schauder D Xin G Nguyen C Wu X Zajac A . CD4(+) T cell help is required for the formation of a cytolytic CD8(+) T cell subset that protects against chronic infection and cancer. Immunity (2019) 51:1028–1042.e4. doi: 10.1016/j.immuni.2019.10.009 31810883 PMC6929322

[12] Hudson WH Gensheimer J Hashimoto M Wieland A Valanparambil RM Li P . Proliferating transitory T cells with an effector-like transcriptional signature emerge from PD-1(+) stem-like CD8(+) T cells during chronic infection. Immunity (2019) 51:1043–1058.e4. doi: 10.1016/j.immuni.2019.11.002 31810882 PMC6920571

[13] Philip M Fairchild L Sun L Horste EL Camara S Shakiba M . Chromatin states define tumour-specific T cell dysfunction and reprogramming. Nature (2017) 545:452–6. doi: 10.1038/nature22367 PMC5693219 28514453

[14] Miller BC Sen DR Al Abosy R Bi K Virkud YV LaFleur MW . Subsets of exhausted CD8(+) T cells differentially mediate tumor control and respond to checkpoint blockade. Nat Immunol (2019) 20:326–36. doi: 10.1038/s41590-019-0312-6 PMC6673650 30778252

[15] Siddiqui I Schaeuble K Chennupati V Fuertes Marraco SA Calderon-Copete S Pais Ferreira D . Intratumoral Tcf1(+)PD-1(+)CD8(+) T cells with stem-like properties promote tumor control in response to vaccination and checkpoint blockade immunotherapy. Immunity (2019) 50:195–211.e10. doi: 10.1016/j.immuni.2018.12.021 30635237

[16] Tsui C Kretschmer L Rapelius S Gabriel SS Chisanga D Knopper K . MYB orchestrates T cell exhaustion and response to checkpoint inhibition. Nature (2022) 609:354–60. doi: 10.1038/s41586-022-05105-1 PMC9452299 35978192

[17] Gautam S Fioravanti J Zhu W Le Gall JB Brohawn P Lacey NE . The transcription factor c-myb regulates CD8(+) T cell stemness and antitumor immunity. Nat Immunol (2019) 20:337–49. doi: 10.1038/s41590-018-0311-z PMC6489499 30778251

[18] Pais Ferreira D Silva JG Wyss T Fuertes Marraco SA Scarpellino L Charmoy M . Central memory CD8(+) T cells derive from stem-like Tcf7(hi) effector cells in the absence of cytotoxic differentiation. Immunity (2020) 53:985–1000.e11. doi: 10.1016/j.immuni.2020.09.005 33128876

[19] Shan Q Hu SS Zhu S Chen X Badovinac VP Peng W . Tcf1 preprograms the mobilization of glycolysis in central memory CD8(+) T cells during recall responses. Nat Immunol (2022) 23:386–98. doi: 10.1038/s41590-022-01131-3 PMC8904300 35190717

[20] Shan Q Zhu S Chen X Liu J Yuan S Li X . Tcf1-CTCF cooperativity shapes genomic architecture to promote CD8(+) T cell homeostasis. Nat Immunol (2022) 23:1222–35. doi: 10.1038/s41590-022-01263-6 PMC9579964 35882936

[21] Johnson JL Georgakilas G Petrovic J Kurachi M Cai S Harly C . Lineage-determining transcription factor TCF-1 initiates the epigenetic identity of T cells. Immunity (2018) 48:243–257.e10. doi: 10.1016/j.immuni.2018.01.012 29466756 PMC5824646

[22] Jameson SC Masopust D . Understanding subset diversity in T cell memory. Immunity (2018) 48:214–26. doi: 10.1016/j.immuni.2018.02.010 PMC5863745 29466754

[23] Masopust D Soerens AG . Tissue-resident T cells and other resident leukocytes. Annu Rev Immunol (2019) 37:521–46. doi: 10.1146/annurev-immunol-042617-053214 PMC7175802 30726153

[24] Wakim LM Woodward-Davis A Bevan MJ . Memory T cells persisting within the brain after local infection show functional adaptations to their tissue of residence. Proc Natl Acad Sci U. S. A (2010) 107:17872–9. doi: 10.1073/pnas.1010201107 PMC2964240 20923878

[25] Loi JK Alexandre YO Senthil K Schienstock D Sandford S Devi S . Corneal tissue-resident memory T cells form a unique immune compartment at the ocular surface. Cell Rep (2022) 39:110852. doi: 10.1016/j.celrep.2022.110852 35613584

[26] Urban SL Jensen IJ Shan Q Pewe LL Xue HH Badovinac VP . Peripherally induced brain tissue-resident memory CD8(+) T cells mediate protection against CNS infection. Nat Immunol (2020) 21:938–49. doi: 10.1038/s41590-020-0711-8 PMC7381383 32572242

[27] Slutter B Van Braeckel-Budimir N Abboud G Varga SM Salek-Ardakani S Harty JT . Dynamics of influenza-induced lung-resident memory T cells underlie waning heterosubtypic immunity. Sci Immunol (2017) 2. doi: 10.1126/sciimmunol.aag2031 PMC5590757 28783666

[28] Borges da Silva H Beura LK Wang H Hanse EA Gore R Scott MC . The purinergic receptor P2RX7 directs metabolic fitness of long-lived memory CD8(+) T cells. Nature (2018) 559:264–8. doi: 10.1038/s41586-018-0282-0 PMC6054485 29973721

[29] Borges da Silva H Peng C Wang H Wanhainen KM Ma C Lopez S . Sensing of ATP *via* the purinergic receptor P2RX7 promotes CD8(+) trm cell generation by enhancing their sensitivity to the cytokine TGF-beta. Immunity (2020) 53:158–171.e6. doi: 10.1016/j.immuni.2020.06.010 32640257 PMC8026201

[30] Peng C Huggins MA Wanhainen KM Knutson TP Lu H Georgiev H . Engagement of the costimulatory molecule ICOS in tissues promotes establishment of CD8(+) tissue-resident memory T cells. Immunity (2022) 55:98–114.e5. doi: 10.1016/j.immuni.2021.11.017 34932944 PMC8755622

[31] Mackay LK Rahimpour A Ma JZ Collins N Stock AT Hafon ML . The developmental pathway for CD103(+)CD8+ tissue-resident memory T cells of skin. Nat Immunol (2013) 14:1294–301. doi: 10.1038/ni.2744 24162776

[32] Sheridan BS Pham QM Lee YT Cauley LS Puddington L Lefrancois L . Oral infection drives a distinct population of intestinal resident memory CD8(+) T cells with enhanced protective function. Immunity (2014) 40:747–57. doi: 10.1016/j.immuni.2014.03.007 PMC4045016 24792910

[33] Zhang N Bevan MJ . Transforming growth factor-beta signaling controls the formation and maintenance of gut-resident memory T cells by regulating migration and retention. Immunity (2013) 39:687–96. doi: 10.1016/j.immuni.2013.08.019 PMC3805703 24076049

[34] Hu Y Lee YT Kaech SM Garvy B Cauley LS . Smad4 promotes differentiation of effector and circulating memory CD8 T cells but is dispensable for tissue-resident memory CD8 T cells. J Immunol (2015) 194:2407–14. doi: 10.4049/jimmunol.1402369 PMC4337487 25637015

[35] Liao W Liu Y Ma C Wang L Li G Mishra S . The downregulation of IL-18R defines bona fide kidney-resident CD8(+) T cells. iScience (2021) 24:101975. doi: 10.1016/j.isci.2020.101975 33474536 PMC7803637

[36] Crowl JT Heeg M Ferry A Milner JJ Omilusik KD Toma C . Tissue-resident memory CD8(+) T cells possess unique transcriptional, epigenetic and functional adaptations to different tissue environments. Nat Immunol (2022) 23:1121–31. doi: 10.1038/s41590-022-01229-8 PMC10041538 35761084

[37] Hirai T Yang Y Zenke Y Li H Chaudhri VK de la Cruz Diaz JS . Competition for active TGFbeta cytokine allows for selective retention of antigen-specific tissue- resident memory T cells in the epidermal niche. Immunity (2021) 54:84–98.e5. doi: 10.1016/j.immuni.2020.10.022 33212014 PMC7856016

[38] Mani V Bromley SK Aijo T Mora-Buch R Carrizosa E Warner RD . Migratory DCs activate TGF-beta to precondition naive CD8(+) T cells for tissue-resident memory fate. Sci (2019) 366. doi: 10.1126/science.aav5728 PMC6939608 31601741

[39] Christo SN Evrard M Park SL Gandolfo LC Burn TN Fonseca R . Discrete tissue microenvironments instruct diversity in resident memory T cell function and plasticity. Nat Immunol (2021) 22:1140–51. doi: 10.1038/s41590-021-01004-1 34426691

[40] Pizzolla A Nguyen THO Smith JM Brooks AG Kedzieska K Heath WR . Resident memory CD8(+) T cells in the upper respiratory tract prevent pulmonary influenza virus infection. Sci Immunol (2017) 2. doi: 10.1126/sciimmunol.aam6970 28783656

[41] Shwetank Abdelsamed HA Frost EL Schmitz HM Mockus TE Youngblood BA . And. Immunol Cell Biol (2017) 95:953–9. doi: 10.1038/icb.2017.62 PMC5698165 28829048

[42] Lee YT Suarez-Ramirez JE Wu T Redman JM Bouchard K Hadley GA . Environmental and antigen receptor-derived signals support sustained surveillance of the lungs by pathogen-specific cytotoxic T lymphocytes. J Virol (2011) 85:4085–94. doi: 10.1128/JVI.02493-10 PMC3126261 21345961

[43] Mackay LK Stock AT Ma JZ Jones CM Kent SJ Mueller SN . Long-lived epithelial immunity by tissue-resident memory T (TRM) cells in the absence of persisting local antigen presentation. Proc Natl Acad Sci USA (2012) 109:7037–42. doi: 10.1073/pnas.1202288109 PMC3344960 22509047

[44] Casey KA Fraser KA Schenkel JM Moran A Abt MC Beura LK . Antigen-independent differentiation and maintenance of effector-like resident memory T cells in tissues. J Immunol (2012) 188:4866–75. doi: 10.4049/jimmunol.1200402 PMC3345065 22504644

[45] Shin H Iwasaki A . A vaccine strategy that protects against genital herpes by establishing local memory T cells. Nature (2012) 491:463–7. doi: 10.1038/nature11522 PMC3499630 23075848

[46] Khan TN Mooster JL Kilgore AM Osborn JF Nolz JC . Local antigen in nonlymphoid tissue promotes resident memory CD8+ T cell formation during viral infection. J Exp Med (2016) 213:951–66. doi: 10.1084/jem.20151855 PMC4886364 27217536

[47] Muschaweckh A Buchholz VR Fellenzer A Hessel C Konig PA Tao S . Antigen-dependent competition shapes the local repertoire of tissue-resident memory CD8+ T cells. J Exp Med (2016) 213:3075–86. doi: 10.1084/jem.20160888 PMC5154944 27899444

[48] Jin Y Xia M Saylor CM Narayan K Kang J Wiest DL . Cutting edge: Intrinsic programming of thymic gammadeltaT cells for specific peripheral tissue localization. J Immunol (2010) 185:7156–60. doi: 10.4049/jimmunol.1002781 PMC3023158 21068400

[49] Wijeyesinghe S Beura LK Pierson MJ Stolley JM Adam OA Ruscher R . Expansible residence decentralizes immune homeostasis. Nature (2021) 592:457–62. doi: 10.1038/s41586-021-03351-3 PMC8057530 33731934

[50] Bilate AM London M Castro TBR Mesin L Bortolatto J Kongthong S . T Cell receptor is required for differentiation, but not maintenance, of intestinal CD4(+) intraepithelial lymphocytes. Immunity (2020) 53:1001–1014 e20. doi: 10.1016/j.immuni.2020.09.003 33022229 PMC7677182

[51] Lauron EJ Yang L Harvey IB Sojka DK Williams GD Paley MA . Viral MHCI inhibition evades tissue-resident memory T cell formation and responses. J Exp Med (2019) 216:117–32. doi: 10.1084/jem.20181077 PMC6314518 30559127

[52] Schenkel JM Fraser KA Masopust D . Cutting edge: resident memory CD8 T cells occupy frontline niches in secondary lymphoid organs. J Immunol (2014) 192:2961–4. doi: 10.4049/jimmunol.1400003 PMC3965619 24600038

[53] Beura LK Wijeyesinghe S Thompson EA Macchietto MG Rosato PC Pierson MJ . T Cells in nonlymphoid tissues give rise to lymph-Node-Resident memory T cells. Immunity (2018) 48:327–38.e5. doi: 10.1016/j.immuni.2018.01.015 29466758 PMC5828517

[54] Anthony SM Braeckel-Budimir NV Moioffer SJ van de Wall S Shan Q Vijay R . Protective function and durability of mouse lymph node-resident memory CD8(+) T cells. Elife (2021) 10. doi: 10.7554/eLife.68662 PMC8213409 34143731

[55] Stolley JM Johnston TS Soerens AG Beura LK Rosato PC Joag V . Retrograde migration supplies resident memory T cells to lung-draining LN after influenza infection. J Exp Med (2020) 217. doi: 10.1084/jem.20192197 PMC7398169 32568362

[56] Suarez-Ramirez JE Chandiran K Brocke S Cauley LS . Immunity to respiratory infection is reinforced through early proliferation of lymphoid TRM cells and prompt arrival of effector CD8 T cells in the lungs. Front Immunol (2019) 10:1370. doi: 10.3389/fimmu.2019.01370 31258537 PMC6587114

[57] Thome JJ Yudanin N Ohmura Y Kubota M Grinshpun B Sathaliyawala T . Spatial map of human T cell compartmentalization and maintenance over decades of life. Cell (2014) 159:814–28. doi: 10.1016/j.cell.2014.10.026 PMC4243051 25417158

[58] Woon HG Braun A Li J Smith C Edwards J Sierro F . Compartmentalization of total and virus-specific tissue-resident memory CD8+ T cells in human lymphoid organs. PloS Pathog (2016) 12:e1005799. doi: 10.1371/journal.ppat.1005799 27540722 PMC4991796

[59] Im SJ Konieczny BT Hudson WH Masopust D Ahmed R . PD-1+ stemlike CD8 T cells are resident in lymphoid tissues during persistent LCMV infection. Proc Natl Acad Sci USA (2020) 117:4292–99. doi: 10.1073/pnas.1917298117 PMC7049149 32034098

[60] Beltra JC Manne S Abdel-Hakeem MS Kurachi M Giles JR Chen Z . Developmental relationships of four exhausted CD8(+) T cell subsets reveals underlying transcriptional and epigenetic landscape control mechanisms. Immunity (2020) 52:825–841.e8. doi: 10.1016/j.immuni.2020.04.014 32396847 PMC8360766

[61] Duckworth BC Lafouresse F Wimmer VC Broomfield BJ Dalit L Alexandre YO . Effector and stem-like memory cell fates are imprinted in distinct lymph node niches directed by CXCR3 ligands. Nat Immunol (2021) 22:434–48. doi: 10.1038/s41590-021-00878-5 33649580

[62] Ozga AJ Chow MT Lopes ME Servis RL Di Pilato M Dehio P . CXCL10 chemokine regulates heterogeneity of the CD8(+) T cell response and viral set point during chronic infection. Immunity (2022) 55:82–97.e8. doi: 10.1016/j.immuni.2021.11.002 34847356 PMC8755631

[63] Bangs DJ Tsitsiklis A Steier Z Chan SW Kaminski J Streets A . CXCR3 regulates stem and proliferative CD8+ T cells during chronic infection by promoting interactions with DCs in splenic bridging channels. Cell Rep (2022) 38:110266. doi: 10.1016/j.celrep.2021.110266 35045305 PMC8896093

[64] Gabriel SS Tsui C Chisanga D Weber F Llano-Leon M Gubser PM . Transforming growth factor-b-regulated mTOR activity preserves cellular metabolism to maintain long-term T cell responses in chronic infection. Immunity (2021) 54:1–17. doi: 10.1016/j.immuni.2021.06.007 34233154

[65] Chen Y Zander RA Wu X Schauder DM Kasmani MY Shen J . BATF regulates progenitor to cytolytic effector CD8(+) T cell transition during chronic viral infection. Nat Immunol (2021) 22:996–1007. doi: 10.1038/s41590-021-00965-7 34282329 PMC9258987

[66] Hu Y Hudson WH Kissick HT Medina CB Baptista AP Ma C . TGF-beta regulates the stem-like state of PD-1+ TCF-1+ virus-specific CD8 T cells during chronic infection. J Exp Med (2022) 219. doi: 10.1084/jem.20211574 PMC9393409 35980386

[67] Ma C Wang L Liao W Liu Y Mishra S Li G . TGF-beta promotes stem-like T cells *via* enforcing their lymphoid tissue retention. J Exp Med (2022) 219. doi: 10.1084/jem.20211538 PMC9393408 35980385

[68] Jansen CS Prokhnevska N Master VA Sanda MG Carlisle JW Bilen MA . An intra-tumoral niche maintains and differentiates stem-like CD8 T cells. Nature (2019) 576:465–70. doi: 10.1038/s41586-019-1836-5 PMC7108171 31827286

[69] Eberhardt CS Kissick HT Patel MR Cardenas MA Prokhnevska N Obeng RC . Functional HPV-specific PD-1(+) stem-like CD8 T cells in head and neck cancer. Nature (2021) 597:279–84. doi: 10.1038/s41586-021-03862-z PMC10201342 34471285

[70] Connolly KA Kuchroo M Venkat A Khatun A Wang J William I . A reservoir of stem-like CD8(+) T cells in the tumor-draining lymph node preserves the ongoing antitumor immune response. Sci Immunol (2021) 6:eabg7836. doi: 10.1126/sciimmunol.abg7836 34597124 PMC8593910

[71] Schenkel JM Herbst RH Canner D Li A Hillman M Shanahan SL . Conventional type I dendric cells maintain a reservoir of proliferative tumor-antigen specific TCF-1(+) CD8(+) T cells in tumor-draining lymph nodes. Immunity (2021) 54:2338–53.e6. doi: 10.1016/j.immuni.2021.08.026 PMC8604155 34534439

[72] Li G Srinivasan S Wang L Ma C Guo K Xiao W . TGF-beta-dependent lymphoid tissue residency of stem-like T cells limits response to tumor vaccine. Nat Commun (2022) 13:6043. doi: 10.1038/s41467-022-33768-x 36229613 PMC9562983

[73] Li Z Tuong ZK Dean I Willis C Gaspal F Fiancette R . *In vivo* labeling reveals continuous trafficking of TCF-1+ T cells between tumor and lymphoid tissue. J Exp Med (2022) 219. doi: 10.1084/jem.20210749 PMC9048291 35472220

[74] Gearty SV Dundar F Zumbo P Espinosa-Carrasco G Shakiba M Sanchez-Rivera FJ . An autoimmune stem-like CD8 T cell population drives type 1 diabetes. Nature (2022) 602:156–61. doi: 10.1038/s41586-021-04248-x PMC9315050 34847567

[75] Skon CN Lee JY Anderson KG Masopust D Hogquist KA Jameson SC . Transcriptional downregulation of S1pr1 is required for the establishment of resident memory CD8+ T cells. Nat Immunol (2013) 14:1285–93. doi: 10.1038/ni.2745 PMC3844557 24162775

[76] Molodtsov AK Khatwani N Vella JL Lewis KA Zhao Y Han J . Resident memory CD8(+) T cells in regional lymph nodes mediate immunity to metastatic melanoma. Immunity (2021) 54:2117–2132.e7. doi: 10.1016/j.immuni.2021.08.019 34525340 PMC9015193

